# A Sixth Mass Extinction? How Linguistic Uncertainty Shapes Our Understanding of the Biodiversity Crisis

**DOI:** 10.1002/ece3.70653

**Published:** 2024-11-28

**Authors:** Lily Linke, Christopher F. Clements

**Affiliations:** ^1^ School of Biological Sciences University of Bristol Bristol UK

**Keywords:** anthropocene, holocene, language, science communication

## Abstract

The term ‘sixth mass extinction’ has become synonymous with the current biodiversity crisis. However, despite a general agreement that current biodiversity declines are severe, no consensus has been reached on whether this constitutes a ‘mass extinction event’, and thus, whether our current situation is comparable to the catastrophic extinction events of deep time. Here, we suggest that our inability to gauge whether the current biodiversity crisis is a mass extinction event may lie less in quantifiable evidence and more in the language used to define such events. We highlight areas of linguistic contention, vagueness and epistemic dispute, and discuss the role of post hoc decision‐making and language in shaping our understanding and communication of biodiversity loss. Our discussion raises larger questions about how we communicate science to the public, funders and other scientists, and how we use language to both shape awareness and leverage action.

## Abstract 2

The term ‘sixth mass extinction’ used for the current biodiversity crisis lacks consensus on its accuracy. The epistemic dispute stemming from this ambiguity underscores the importance of clear communication in shaping public awareness and spurring action regarding biodiversity loss.

## 1 Introduction

It seems indisputable that the earth is currently experiencing an anthropogenically driven ecological crisis (Davis, Faurby, and Svenning 2018; Leakey and Lewin 1996; Singh 2002; Turvey and Fritz 2011). Human interference has been detrimentally affecting local biodiversity since at least the late Pleistocene epoch over 11,700 years ago (Diamond 1989), while global industrialisation and human range expansion have exacerbated this effect with multifaceted threats including overexploitation, climatic change and habitat fragmentation and degradation (Lande 1998; WWF 2022). The current magnitude of biodiversity loss has raised the question: Are we in the midst of a ‘mass extinction’ event?

In the past 4.1 billion years, up to 18 ‘mass extinction’ events have been identified through fossil record analysis (Bambach 2006; Marshall 2023) with five of these, the ‘Big Five’, being widely recognised as the most catastrophic (Racki 2019). Since the mid‐1990s, scientists have been referring to the current period of biodiversity change as an extinction event (Bocchi et al. 2022), drawing parallels between the end‐Permian extinction (the ‘Great Dying’), which wiped out an estimated 95% of species on Earth (Erwin 1989) and current rates of global environmental change (NOAA 2020; Penn and Deutsch 2022). These factors include declining ocean O_2_ and pH, rising global temperatures and high heat‐trapping gas emissions (Cui and Kump 2015; Jurikova et al. 2020). Furthermore, the current rate of species extinction has been put between 100 and 1000 times the background rate across recent geological time (Ceballos et al. 2015; Ceballos and Ehrlich 2018; Pimm et al. 2014). However, whether this constitutes a mass extinction event or not remains contentious, as no unifying definition of mass extinction exists. We argue that our inability to categorise the present ecological crisis is in part driven by the vague terminologies in extinction literature and that more clearly defining these terms will help put the current biodiversity crisis in context. Vagueness in definition can have profound real‐world implications, with examples from other disciplines (Somerville and Hassol 2011; Williams 2018) showing how vague language can be leveraged to serve an agenda through the spread of misinformation and pseudoscience. In this paper, we will give an overview of the linguistic theory, which may be applied to the conservation literature, deconstruct well‐recognised definitions of mass extinctions to analyse the role of linguistic imprecision and identify the impact of linguistic choices on mobilisation of policymakers and the public. We propose the introduction of linguistic theory as a tool to inform conservation practice and decision‐making and provide a framework by which others may be able to identify and remedy vagueness in their own work.

## 2 Epistemic Uncertainty vs. Linguistic Uncertainty

In 1994, Williamson determined that uncertainty within scientific debate falls under two categories: epistemic and linguistic (Williamson 1997). Epistemic uncertainty arises due to a lack of data or the use of interpolation and extrapolation. It can be rectified through more extensive data collection and increasing modelling accuracy. For example, Bambach, Knoll, and Wang (2004) analysed the fossil record between 400 and 200 Ma and determined that origination rates decreased in the late Devonian and end‐Triassic periods, but Foote (Foote 2003) and Kocsis et al. (2019) found that origination rates increased during this same period. This uncertainty is derived from the incompleteness in the fossil record being corrected for by differing modelling techniques and is therefore epistemic. The literature covers epistemic (Jablonski 1989; Myers 1993; Newman 1997) and philosophical (Bocchi et al. 2022; Cafaro 2015; Santana 2019) uncertainty surrounding the mass extinction debate extensively. However, the role of linguistic uncertainty has been primarily neglected.

‘Linguistic uncertainty’ is derived from the use of natural language in the place of, or where there may be a lack of, unequivocally precise terminology (Regan, Colyvan, and Burgman 2000). The linguistic uncertainty in question is typically ‘vagueness’, occasionally used interchangeably in the literature with ‘fuzziness’ (Klir 1987). Vagueness is unanimously understood to be ever‐present, but the question of its functionality has been dividing schools of thought in philosophy and computing for decades (Russell 1923; Seising 2008). Drawing on general philosophical and linguistic literature, we find there to be two key philosophies on the origins of vagueness. As the literature is so diverse, it is important to clarify that these descriptions cannot encompass all nuance available but intend to provide an accessible overview of the two parties. An epistemologist would argue that linguistic uncertainty stems from a lack of context, or as a placeholder for things we do not or cannot know (Schiffer 1999). They put forward that vagueness is not something that naturally exists, it emerges from an inability to be more accurately descriptive. An epistemologist would argue that the linguistic ‘fuzziness’ arising from attempts to define mass extinction events is a *failure* in precision (Williamson 1997) and may stem directly from epistemic uncertainty or a lack of appropriate context. One may believe that there is a critical value at which an event becomes a mass extinction, we simply do not know what that value is.

The opposing philosophy, adopted by many supervaluationists (Varzi 2007; Weatherson 2003), acknowledges the role of context and epistemic uncertainty but instead argues that vague objects do exist. Therefore, vague descriptions can have a level of truth. For example, a ‘heap’: The Sorites Paradox poses—how many grains of sand does it take to form a heap? The paradox emerges from the logic that if one grain of sand is not a heap, so two grains of sand are not a heap, and three grains are not a heap; at no point is *n* + 1 grains of sand a heap and so a heap of sand does not exist. Should one work backwards from the presumption that n grains of sand *is* a heap, then *n* − 1 grains of sand is a heap, *n* − 2 grains of sand is a heap, etc., until 1 grain of sand must also be a heap. In this way, any number of grains of sand is a heap but also no number of grains of sand is a heap. Weber and Colyvan (2010) raised that terminology lacking in sharp boundaries, for example, vague predicates such as ‘endangered’, leave conclusions vulnerable to the Sorites Paradox. If we were to isolate species extinction as the sole predictor of a mass extinction event, a grain of sand would represent one species extinction and ‘heap’ in the sorites paradox could be replaced with the term ‘mass extinction’, thereby categorising a mass extinction as a vague object.

## 3 The Theory of Vagueness

The implications of vagueness are not arising solely from the phrase ‘mass extinction’. Attempting the outline the precursors for a mass extinction event also leads to vagueness. This paper is also concerned with the use of vague predicate adjectives and resulting vague terminologies and definitions. A common example is ‘tall’ (Solt 2011). According to the Sorites' logic, there can be no height at which a person is tall. As a stand‐alone adjective, ‘tall’ is ostensibly meaningless; much of its meaning emerges from the context in which it is applied, for example, ‘tall’ for an 8‐year‐old conjures a very different image to ‘tall’ for a 40‐year‐old. However, even with context present, there is plenty of room for differing interpretations depending on a multitude of other qualifiers (a person's nutrition, region of origin, etc), including the experiences of the audience receiving the information. Therefore, there are upper and lower bounds of a *reasonable* interpretation for ‘tall’ (Gross 2009; Lawry 2008; Sorensen 2001). Descriptors such as ‘widespread’, ‘far above’, ‘substantial’ are being used to describe mass extinction events in the literature. Each descriptor has a *reasonable* interpretation with upper and lower bounds when employed without adequate clarification. The acknowledgement of these upper and lower bounds, called supervaluation, is where we may recognise that there are ‘borderline cases’ within truthfulness; global biodiversity loss may be justifiably considered ‘substantial’ and ‘not substantial’ by two different parties according to their respective understandings of the word ‘substantial’, neither party are categorically wrong.

By recognising borderline cases, we must acknowledge that some terms in context are vaguer, or less explicitly truthful than others. This is where it is valuable to entertain the concept of truth values. In classical logic, truth values exist as a pair (0, 1) in which 1 represents truth and 0 represents falsity. The epistemologist may argue for the imposition of a ‘sharp boundary’ at which a case switches from true to false (and vice versa), for example, we have reached ‘sixth mass extinction’ status at 75% global species extinction but not at 74%. In many‐valued logic, this set (0, 1) is generalised to include states beyond complete truth and falsity. For example, paraconsistent logics allow a state corresponding to a paradoxical sentence being simultaneously true and false (e.g., the Sorites paradox, known as a dialetheia), while fuzzy logic allows a proposition in which a degree of truth is found at a value between 0 and 1, which may be seen as a degree of membership to the truth (Unwin 1986), for example, should a biodiversity crisis meet only one out of 10 requirements for mass extinction status, we might say that the term ‘mass extinction’ has a truth value of only 0.1 when applied to that crisis.

## 4 Different Types of Vagueness

Understanding relevant modes of logic is necessary to inform an understanding of vagueness, but there are also practical steps that may be taken to ‘cope’ with it. Regan, Colyvan, and Burgman (2002) deconstruct vagueness into categories: context‐dependence, ambiguity, underspecificity and indeterminacy of theoretical terms (Table 1). The term ‘mass extinction’ is context‐dependent, ambiguous, and in some cases indeterminate. However, it is counterproductive to define it as a paradoxical term as this renders it inoperable. It is important to recognise proposed remedies for different types of vagueness to allow the terminologies to be useful. The ambiguity of ‘biodiversity,’ for example, is crucial to consider in discussions of mass extinction as it represents the things we are concerned about disappearing. The implications of vagueness have been raised numerously regarding the term ‘biodiversity’ (Kaennel 1998; Pence 2023). The term itself exists as a contraction of ‘biological diversity’ as popularised by Rosen in 1985 for the National Forum on BioDiversity (Oksanen and Pietarinen 2004). Due to the nature of its synthesis, the word has come to be associated with a multitude of definitions and associations across various disciplines. It is sometimes used only in reference to the number of species or the richness of species in an area, it may also be used to encompass all features of the varieties and configurations of all living organisms considering everything between genetic diversity and ecosystem diversity. Faith (Faith 2023) poses that ‘(the) increased popularity of the term among academics has amounted to decreased clarity of the term’. It has, perhaps, come to be best understood as a collection of tools used to determine biological (or biologically adjacent) variety rather than a precise terminology. Meinard, Coq, and Schmid (Meinard, Coq, and Schmid 2019) argue that conservationists have an obligation to stop promoting a term which impedes conservation efforts through vagueness. Instead, they propose the introduction of a ‘dynamic definition’ for biodiversity, one that is open to criticism and modification. Bargheer (2024) outline that this vagueness is not a ‘design flaw’ but an intended feature that does not lend itself well to guiding scientific research or unified mobilisation. This aligns with the supervaluationist's philosophy that vagueness is not a fault, it merely requires recognition to be approached appropriately for the context.

**TABLE 1:** Categories of vagueness, their respective definitions, and relevant examples in conservation literature.

Category of Vagueness	Definition	Example
Context‐dependence	Underdetermination, there is not enough relevant context for the receiver to evaluate the meaning of a term. Context can be both situational i.e., the purpose of the statement, and provided within the terminology i.e., qualifiers given within a sentence	The population is small
Ambiguity	Overdetermination, there are multiple correct definitions available under an umbrella of the same terminology	Biodiversity
Under‐specificity	Underdetermination, a term that remains unclear following the addition of context because the predicate itself is imprecise	‘Amount’ of species extinction
Indeterminacy of theoretical terms	An unstable grounding for the emergence of a word leading to an evolution of its meaning over time	Species

*Note:* Adapted from Regan, Colyvan, and Burgman (2002).

Vagueness has been discussed in a limited capacity regarding extinction events (Regan, Colyvan, and Burgman 2000) despite large discrepancies in the language present in the literature. Recognition of vagueness in extinction literature largely refers to defining categories of endangerment (Cheung, Pitcher, and Pauly 2005; Regan, Colyvan, and Burgman 2000). The focus of these studies has been epistemic vagueness, but the proposed remedy is consistent via the linguistic approach too. Cheung, Pitcher, and Pauly (2005) move to introduce a ‘fuzzy expert system’ in which degrees of membership to ‘intrinsic vulnerability to extinction’ range from 0 to 1 within the categories of very high, high, moderate and low. This methodology stems from the aforementioned truth values of many‐valued logic. It is worth considering that this methodology may be upscaled for application to defining a mass extinction event. For example, Bambach, Knoll, and Wang (2004) refer to the Late‐Devonian and End‐Triassic ‘extinctions’ as mass ‘depletions’ due to their leading cause being low origination rates rather than species death. However, as the literature moves to recognise that mass extinctions are not a homogenous group of events (Bocchi et al. 2022) and are not always defined solely by the quantity of species extinction (Cerini, Childs, and Clements 2023; Naeem 2002), it may be argued that this linguistic distinction is obsolete. The term ‘depletion’ is just as ambiguous as ‘extinction’. Therefore, when we consider what critical point a mass depletion becomes a mass extinction event (Bambach, Knoll, and Wang 2004), perhaps it would be more useful to observe a mass depletion as a level of membership to, or indicator of, a mass extinction event rather than a separate subgenre of disaster.

## 5 Tackling Vagueness in Specific Cases of Defining Mass Extinctions

When vagueness goes unacknowledged, attempts to define a mass extinction are biased by the author's intention or the receiver's experience. In this way, it is necessary to evaluate the functionality (or futility) of vagueness present in specific definitions in the literature.

### 5.1 Sepkoski (1986)

A mass extinction may be defined as … ‘any substantial increase in the amount of extinction (i.e., lineage termination) suffered by more than one geographically wide‐spread higher taxon during a relatively short interval of geologic time, resulting in an at least temporary decline in their standing diversity’.

Sepkoski's was one of the earliest attempts to provide a general definition for periods of accelerated extinction identified in the fossil record. This definition leaves linguistic room for researcher subjectivity in six key areas: (1) how to quantify the ‘amount’ of extinction, (2) how much extinction is ‘substantial’, (3) how to classify a taxon as ‘widespread’, (4) what counts as a ‘higher‐taxon’, (5) what qualifies as a ‘relatively short’ geological time interval and (6) more broadly what is meant by an ‘at least temporary decline’ in diversity? These terms fall under the umbrella of ‘vague quantifiers’ (Solt 2011), which typically have unknowable boundaries for application. Therefore, any attempt to utilise this definition in a context other than for Sepkoski's original application will yield an answer guided by the researcher's interpretation and intention. It is clear that the terms ‘substantial’, ‘widespread’ and ‘relatively short’ fall prey to the same underspecificity and context‐dependence issues outlined in cases such as the aforementioned ‘tall’. However, it is necessary to qualify that despite breaking the definition apart to identify individual cases of vagueness, to recognise the context‐dependent quality of meaning we can only functionally evaluate the vagueness of these predicates with consideration of the definition as a whole. The interplay between vague predicates forms a novel collection of specifications. Sepkoski's use of the vague term ‘substantial’ is specified through further context given within the definition; he refers to a ‘substantial increase’ *in* the amount of extinction *of* a widespread taxon *for* an amount of time. However, the presence of context cannot remedy vagueness if each qualifying term is also underspecific. Bambach, Knoll, and Wang (2004) evaluate the vagueness of ‘higher taxa’ and ‘geographically widespread’, identifying the benefits of leaving things unsaid; it would be both ‘arbitrary’ and ‘limiting’ to specify either the number of taxa or the taxonomic level, and there should not be a requirement for extinction to be uniform or global. This defence falls in alignment with the supervaluationist ideology that vague objects exist. If a researcher considers a mass extinction to be a vague entity, then vague descriptors are truthful components of its definition. Sepkoski states that a mass extinction is ‘any’ event that falls within the bounds of the vague terminologies within the definition. Through tabulating data from Sepkoski's marine genus database (Sepkoski 2002), Bambach, Knoll, and Wang (2004) determined 18 intervals in the Phanerozoic that are technically classed as mass extinctions under Sepkoski's definition, not just the ‘Big Five’ that have been so universally accepted. This implies that there is a collection of borderline cases for the phrase ‘mass extinction’ and it is, therefore, a vague term. Marshall (2023) outlined that Sepkoski (Raup and Sepkoski 1982) aimed to determine whether the variability in extinction intensities could be adequately explained by a single distribution, thereby suggesting a unified class of explanation, and not to synthesise a definition for predictive or modern application. Sepkoski (1986) states himself that the linguistic imprecision was designed to reflect epistemic uncertainty regarding events of accelerated extinction. This highlights the importance of the recognition alone of vagueness as a valuable technique because it may not need to be remedied. Vagueness serves a purpose within Sepkoski's intention. It is only when applied in the place of a quantitative hypothesis, that this same vagueness impedes definition.

Despite these prerequisites, the definition is abundantly present in modern coverage of the sixth mass extinction. By insisting on the usage of the term ‘mass extinction’ to describe the present crisis, we call for a definition that aligns previous extinction events with the current environment despite the data, timescale and monitoring techniques available to us being so vastly different. It is, therefore, necessary to evaluate the validity of the use of Sepkoski's overview for defining extinction in the Anthropocene. Critics argue that Sepkoski's vague quantifiers insist on the imposition of sharp boundaries for this definition to be presently functional. Padian (2018) has termed the definition ‘inoperably vague’. Padian also argues that the fuzzy language has rendered the study of mass extinction rife with imprecision and pushes to replace the term entirely with ‘diversity crisis’ (which arrives with its own set of qualifiers). Consistent with Padain's criticisms, the philosopher Frege aligns vagueness with negligence (Sorensen 2001): ‘One haphazardly introduces a necessary condition here, a sufficient condition there, but fails to supply a condition that is both necessary and sufficient’. When approaching the present biodiversity crisis, armed with this definition, a researcher will certainly find that it lacks terminologies which are both necessary and sufficient.

An alternative standpoint is that the flexibility of Sepkoski's definition challenges the assumption that mass extinctions must be a homogenous group of events (Bambach, Knoll, and Wang 2004), and it is therefore suitable as a general definition. This aligns with a form of optimistic supervaluationism, in which one would hold the belief that, instead of becoming stuck with a definition as time passes, we may reevaluate meaning in the light of new information and interests. This is often referred to as the open texture of language (Regan, Colyvan, and Burgman 2002). Akin to calls for a ‘dynamic definition’ for biodiversity, this may be considered a dynamic definition for a ‘mass extinction event’. It is convincing that the vagueness of Sepkoski's definition suited its intended purpose. However, it is not appropriate, in its original form, to take on a quantitatively predictive or presently functional quality.

### 5.2 Barnosky et al. (2011)

75% or more of species become extinct within 2 million years

A more ‘conventionally’ (Rull 2022) applied definition was initially proposed by Barnosky et al. (2011). Barnosky argues that his definition is conservative, following the framework of Sepkoski's (1986) and based on Jablonski's (Jablonski and Chaloner 1994) estimation that over 75% of *all* species went extinct at each mass extinction interval within the Big Five. The need for technical language is eliminated by the use of numerical value. The vagueness of the term ‘substantial amount’ of extinction in Sepkoski's definition is reconciled through Barnosky's use of a percentage and ‘relatively short’ geological time frame, ‘less than 2 million years’. This definition may be favoured by the epistemologist, who believes in the existence of precise boundaries, left unidentified by vague terminologies (Williamson 1997).

However, the intermediate level of specificity may hinder both definition and conservation efforts. The introduction of a specific threshold could mask the severity of biodiversity loss (Padian 2018); what does a species loss of 73%, for example, indicate? The replacement of vague terminology with sharp definitions increases the ease with which an event may be categorised as a mass extinction but not necessarily the accuracy. If the definition is taken for application in a research capacity where the audience are ‘informed’ readers, it may be valuable to increase the specificity of 75% species loss to include the number of standard deviations within which an upper and lower boundary for definition of an extinction event may be classified. The imposition of graduated boundaries is supported as an approach to vagueness by fuzzy set logic (Center and Verma 1998; Zadeh 1965) and may be applied through the utilisation of the ‘fuzzy expert system’ (Cheung, Pitcher, and Pauly 2005). Cheung et al. propose the introduction of an index to represent the extinction vulnerability of marine fishes to fishing by using life histories and ecological characteristics. They recognise that vagueness in our understanding of ecological characteristics and intrinsic vulnerability statuses of marine fishes is impeding efforts to categorise extinction risk and therefore, complicates marine conservation efforts. As there is no precise definition of what a ‘large fish’ is despite size being a contributing factor to extinction vulnerability, it is instead useful to consider different maximum body sizes to have a degree of membership to small, medium or large, for example, a fish of maximum length 68 cm may have a 0.7° membership to ‘medium’ and a 0.3° membership to ‘large’. To apply this logic to the present issue, a 75% species extinction may indicate a high degree of membership to a mass extinction event instead of being a threshold for one.

It is further qualified within Barnosky et al.'s paper that extinction rates must accelerate relative to origination rates to reach this extinction percentage, though this is not outlined in the overarching definition. The importance of the inclusion of origination rate in extinction calculations cannot be understated (Alroy 2008) and this omission from the keynote message has led to the criticism that there is ‘no focus’ on origination rates (Padian 2018). In this way, it is valuable to consider the impact of vagueness as an outcome of the absence of appropriate qualifiers, not only the presence of vague qualifiers. In ecology and conservation literature, it has been argued that focussing on ‘species loss’ *specifically*, draws attention away from other indicators and precursors of species extinction, and therefore extinction events (Naeem 2002), such as the erosion of genetic diversity or declines in the health and resilience of populations (Cerini, Childs, and Clements 2023), potentially leading to extinction debt. Jablonski et al. (1997) outlines that two of the ‘Big Five’ may have been the outcome of a drop in origination rates rather than a rise in extinction rates.

In terms of its functional predictive quality, it has been highlighted in all three papers utilising this definition (Barnosky et al. 2011; Gross 2022; Rull 2022) that the current extinction rates *are* higher than those that carried the previous five events to mass extinction and therefore, should these rates remain the same or accelerate, another mass extinction event may occur within the next three centuries. The implication here is either that a sixth mass extinction has not begun until that 75% extinction boundary has been reached, or that a mass extinction may only be viewed as the ‘end‐state’ of an ecological crisis. Researchers using this definition do not consider a ‘mass extinction’ a vague entity but a critical stage beyond a threshold. The epistemic conclusion drawn by researchers utilising this definition (Barnosky et al. 2011; Gross 2022; Rull 2022) is that current species loss ‘does not yet qualify as a mass extinction in the palaeontological sense of the Big Five’. Note the researchers' qualification in the ‘palaeontological sense’. This distinction has also been highlighted by Currie (2019) as a possible linguistic issue in communicating aims across biological disciplines. Certain ambiguous terms leave respective definitions within the realm of one discipline and take on a different definition as they cross into the next. To elaborate, Currie outlines that paleobiologists are concerned with ‘mass extinctions’ while evolutionary biologists study ‘major transitions’. He argues that palaeontology is ‘phenomena‐led’, which aligns with Barnosky's definition of a mass extinction as an end‐state. Contrastingly, evolutionary biology is ‘theory‐led’, which may be better aligned with Sepkoski's general definition. The present ecological crisis is a multidisciplinary concern and therefore the language used to define one should be unifying.

As linguistic interpretation is influenced by audience experience, it is also valuable to evaluate the function of this emphasis on species extinction in more publicly accessible literature. Barnosky's definition has been adopted more frequently than others in media coverage such as BBC News Climate and Science (Hughes 2022) and the Natural History Museum website (Begum 2021). If the applicative purpose of the definition is to generate public concern, it could be argued that the use of more targeted language and accessibly expressed numerical value becomes not only necessary but advantageous. Keeping the public informed facilitates behavioural change on one level but also leads to an increase in societal support for policymakers to direct more resources to prevent an ecological crisis (Khatibi et al. 2021). As it has been hypothesised that public interest in species extinctions may lead to an increase in donations to conservation organisations (Clements 2013), perhaps increased specificity of language more efficiently directs NGO funding.

### 5.3 Ceballos et al. (2015)

The oft‐repeated claim that Earth's biota is entering a sixth “mass extinction” depends on clearly demonstrating that current extinction rates are far above the “background” rates prevailing between the five previous mass extinctions.

That is, the current rate of species loss must be substantially greater than the calculated average extinction rate between The Big Five. This claim was synthesised for modern application upon the presumption that the Big Five are universally agreed to be mass extinction events. By employing the term ‘mass extinction’ for the current period, we directly conflate the modern biodiversity crisis with the crises of the paleontological past. However, it appears that the literature does not provide a comprehensive nor unanimously agreed‐upon definition of a mass extinction for this conflation to be useful. As the ‘Big Five’ represent the largest drops in the Phanerozoic marine fossil record since the early‐Ordovician period and not the total vertebrate fossil record, is it entirely justifiable to broadly coin them ‘The’ Big Five, or would it be more accurate to term them ‘A’ big five (Marshall 2023)? Alroy (Alroy 2008) stated that referring to previous mass extinctions as the big one, three or five is purely a ‘matter of taste’ and Bambach has identified up to 64 possible extinction ‘events’. The number of events that may be classed as ‘mass depletions’ as opposed to ‘mass extinctions’, for example, is still a highly controversial topic (Cowie et al. 2022; Marshall 2023). The uncertain status of prior extinction events leaves a novel definition prone to the same vagueness introduced to fill epistemic gaps historically. However, despite this conflation raising adjacent conceptual issues, it does not increase the vagueness of the definition within the context of the assumption that the Big Five theory is valid.

It is also necessary to consider the implications of the vague quantifier ‘far above’. Beginning with an understanding that vagueness is context‐dependent, it may be argued that a vertebrate extinction rate 100 times higher than the background rate (Ceballos et al. 2015) is indisputably *far above*. We need not consider how the flexibility of the term may impact the endeavour to define mass extinctions as a group, as Ceballos et al. only intend to determine the status of the current crisis concerning an established group of prior crises. The language employed is internally referential, and all necessary context is present.

The data utilised for this calculation was compiled from the ‘extinct’ and ‘possibly extinct’ categories on vertebrate species in the 2014 IUCN Red List, which is the same source drawn upon by Barnosky et al. (2011), Gross (2022) and Rull (2022) for the *same* epistemic conclusion on extinction rates but a *different* stance on the imminence of the sixth mass extinction. When this same data is applied to Ceballos' definition, the criteria are met for a sixth mass extinction now. This is a prime example of how discrepancies in definition distract from the severity of the developing crisis. This is not a criticism of the researchers' intention but an undesirable side effect. It is valuable to acknowledge that Barnosky coauthored Ceballos' 2015 paper. It may be inferred that the responsibility to communicate the severity of the extinction figures unifies the research in the same way that the fuzzy language divides conclusions.

McCallum (2015) provides a qualifier in a similar vein as Ceballos et al. (Ceballos et al. 2015; Ceballos, Ehrlich, and Raven 2020; Ceballos and Ehrlich 2018), arguing that ‘the fraction of species recorded as extinct presently should be proportional to extinctions at the K‐Pg boundary for biodiversity losses to align with mass extinction scale’. Ceballos et al. (2015), and McCallum's (2015) definition may be more applicable than Barnosky et al.'s (2011) in research circles for current and predictive identification of a novel extinction event due to the inclusion of rate analysis allowing for ongoing monitoring. The benefit of drawing specific attention to extinction rates and their catalysts has been reasonably well explored in conservation literature, for example, dietary specialisation is associated with higher extinction rates (Winfree 2010). In this way, identifying populations exhibiting factors associated with accelerated extinction risk adds to the functional predictive quality of the definition; it also directs attention towards areas in which it is not too late for intervention. This definition would lend itself well to the aforementioned ‘fuzzy expert system’ (Cheung, Pitcher, and Pauly 2005).

In the more recent papers authored by Ceballos et al. (Ceballos, Ehrlich, and Raven 2020; Ceballos, Ehrlich, and Dirzo 2017), the term ‘biological annihilation’ has been used in reference to the ‘ongoing’ sixth mass extinction. The use of ‘annihilation’ harnesses the reach of sensational language within the public eye. Sensational language is any language intended to produce a startling impression and has been shown to increase the reach of an article (Ali et al. 2019). A Guardian article referencing Ceballos' term ‘biological annihilation’ published in July of 2017 preceded a notable Google search spike of the term ‘sixth mass extinction’ in the United Kingdom (Google Trends). The phrase has also been adopted by The New York Times in 2017 (‘Era of “Biological Annihilation” Is Underway, Scientists Warn’) and CNN (Sutter 2017). The scientific accuracy of applying this term is unclear and may add to Brigg's (Briggs 2016) criticism of Ceballos' ‘exaggeration literature’ for ‘misleading’ the public. Despite this, the prevailing conclusion appears to be that the importance of communicating the severity of the current biodiversity crisis cannot be overstated (Lees et al. 2020) and that the prevailing impact of the literature should be to stop the government's ‘laissez‐faire’ attitude towards conservation efforts and mobilise the public to push for a more definite solution. When public understanding is guided by vagueness, policymakers can ‘get away’ with a noncommittal approach to conservation. In Williams's (2018) investigation into ‘The Impact of Vague Legislation on Policy’, he identifies vague predicates and qualifiers such as ‘reasonably’ and ‘vulnerable’ in the 1996 UK Housing Act. These terminologies complicated housing access to homeless people by shifting with differing interpretations of vulnerability. In the same way, vague definitions of ecological crises can ‘enable bureaucratic discretion’ (Williams 2018) when guiding legislative efforts. If we cannot identify vague language, we cannot analyse nor remedy its impact.

## 6 Conclusions and Future Directions

Utilising linguistic theory, we have identified modes of logic such as supervaluationism and epistemology and their roles in conservation literature. This has informed an investigation into the shortcomings of the endeavour to define, and communicate conclusions on, mass extinction events.

There is little to no acknowledgement in the current literature of the impact of vague language on the evaluation of a novel mass extinction event and the subsequent allocation of conservation resources. It would be valuable to synthesise a compendium under the umbrella of a general definition upon which a consensus has been reached, in the same vein as Sepkoski's but with the omission of vague quantifiers, and for modern application. We cannot define a novel event with the same language used to define previous mass extinction events under such a differing scope of context and timeline. It would also be valuable to analyse the impact of fuzzy language and categories on both research and publicly led conservation beyond the depth limited by the scope of this review.

Furthermore, a clear set of qualifications would be necessary alongside a general definition as there is not currently a definition which acknowledges the significance of range contraction and population dynamic shifts in the evaluation of a sixth mass extinction despite this being stressed in the body of most of the literature. The definition synthesised by Ceballos et al. with McCallum's qualifier (Ceballos, Ehrlich, and Raven 2020; Ceballos, Ehrlich, and Dirzo 2017; Ceballos and Ehrlich 2018) is the most effective attempt as it has minimised the use of vague quantifiers and takes on a dynamic quality while still acknowledging the value of historical data. Unified with an epistemic conclusion supported in other literature, it is more convincing that the earth is already in the midst of the newest mass extinction event.

However, the decision of whether the current biodiversity crisis constitutes a mass extinction is one which is made post hoc, as the threshold for which we define a ‘mass extinction’ is inherently relative, not absolute. As such, setting a threshold which ‘creates’ a sixth mass extinction (or not) becomes a subjective and not a quantitative choice, subject to the whims and vagaries of researcher (and media) bias. This highlights an issue in the communication of science more broadly—the role of language in shaping the public's understanding of current issues and how language can leverage (or lose) public support. Examples abound in the scientific literature and general media, from the communication of the climate crisis to how GM crops are perceived by the public. This nontrivial and nebulous issue combines elements of science, philosophy and politics, and is a subject which the majority of researchers are underprepared to tackle.

## Author Contributions

**Lily Linke:** conceptualization (equal), writing – original draft (lead), writing – review and editing (equal). **Christopher F. Clements:** conceptualization (equal), supervision (lead), writing – review and editing (equal).

## Conflicts of Interest

The authors declare no conflicts of interest.

## Data Availability

No new data were created or analysed in this study. Data sharing is not applicable to this article.
